# “New” metastases are associated with a poorer prognosis than growth of pre-existing metastases in patients with metastatic breast cancer treated with chemotherapy

**DOI:** 10.1186/s13058-015-0657-1

**Published:** 2015-12-09

**Authors:** Christopher Twelves, Javier Cortes, Peter A. Kaufman, Louise Yelle, Ahmad Awada, Terri A. Binder, Martin Olivo, James Song, Joyce A. O’Shaughnessy, Maria Jove, Edith A. Perez

**Affiliations:** Section of Oncology and Clinical Research, Leeds Institute of Cancer and Pathology, University of Leeds and Leeds Teaching Hospitals Trust, Leeds, UK; Department of Oncology, Vall d’Hebron Institute of Oncology, Barcelona, Spain; Department of Oncology, Ramon y Cajal University Hospital, Madrid, Spain; Section of Hematology/Oncology, Norris Cotton Cancer Center, Dartmouth-Hitchcock Medical Center, Lebanon, NH USA; Department of Medicine, University of Montreal, Montreal, QC Canada; Medical Oncology Clinic, Medicine Department, Jules Bordet Institute, Université Libre de Bruxelles, Brussels, Belgium; Oncology Imaging, Eisai Inc., Woodcliff Lake, NJ USA; Oncology PCU, Clinical Development, Eisai Inc., Woodcliff Lake, NJ USA; Department of Medical Affairs, Eisai Inc., Woodcliff Lake, NJ USA; Department of Medical Oncology, US Oncology, Texas Oncology-Baylor Charles A. Sammons Cancer Center, Dallas, TX USA; Division of Hematology/Oncology, Department of Cancer Biology, Mayo Clinic, Jacksonville, FL USA; St James’ Institute of Oncology, St James’ University Hospital, University of Leeds, Bexley Wing, Level 4, Beckett Street, Leeds, UK

## Abstract

**Introduction:**

Progression-free survival (PFS) and overall survival (OS) endpoints often only weakly correlate. This analysis investigates how different progression events impact on OS, using data from two phase 3 studies with eribulin in women with advanced/metastatic breast cancer (MBC).

**Methods:**

In Study 301, 1102 women with ≤2 prior chemotherapies for advanced/MBC were randomized to eribulin mesylate (1.4 mg/m^2^ on days 1 and 8 every 21 days) or capecitabine (1.25 g/m^2^ twice daily on days 1–14 every 21 days). Study 305/EMBRACE enrolled 762 patients following two to five prior chemotherapies for advanced/MBC, randomized to eribulin (as above) or treatment of physician’s choice. We analyzed OS and PFS post hoc for patients whose disease progressed due to development of “new” metastases, growth of pre-existing lesions, and patients with no reported disease progression.

**Results:**

In both clinical studies, development of new metastases was associated with an increased risk of death (*p* < 0.0001). The time to development of new metastasis or death was significantly longer with eribulin than the comparator in Study 305 (*p* = 0.0017), but not in Study 301 (*p* = 0.46). Significantly longer OS was observed in the eribulin compared with the comparator arm for the new metastases subgroup in Study 301 (*p* = 0.008), but not in Study 305 (*p* = 0.16), compared with other progression subgroups.

**Conclusions:**

Patients with MBC progressing with new metastases have a worse prognosis than those whose disease progresses due to growth of existing lesions or patients with no reported disease progression. These findings have potentially important implications for the interpretation of clinical study data and clinical practice.

**Trial registration:**

ClinicalTrials.gov registration IDs: Study 301: NCT00337103; Study 305: NCT00388726.

## Introduction

Overall survival (OS) has been regarded as the gold-standard endpoint in phase 3 cancer studies since the 1980s when it replaced objective response rate (ORR) as the principal endpoint for studies supporting anticancer drug approval [1, 2]. OS benefits can, however, be difficult to assess because they may require a large patient population and a prolonged follow-up period to demonstrate a statistically significant difference [3, 4]. Additionally, in clinical studies evaluating therapy earlier in the course of metastatic disease, OS may be strongly influenced by subsequent lines of therapy [5, 6]. Many phase 3 oncology studies, including those in metastatic breast cancer (MBC), have therefore used alternative primary endpoints (also known as intermediate endpoints) such as progression-free survival (PFS) [3]. Use of PFS markedly reduces the number of patients necessary to establish a statistically significant benefit, particularly when the time between the progression event and death may be relatively long [4] or when there is extensive crossover [7].

Despite widespread use in clinical trials, many intermediate endpoints are poorly defined or have varying definitions in the literature, making it difficult to consistently interpret treatment effects and reliably perform cross-trial comparisons. The heterogeneity of definitions for endpoints has triggered the recent DATECAN (Definition for the Assessment of Time-to-Event Endpoints in CANcer trials) initiative, aimed at standardizing consensus definitions of endpoints for multiple cancer sites, including breast cancer [8].

Meta-analyses of intermediate endpoints, such as PFS, have suggested that these are often not reliable surrogate endpoints for OS in MBC [3]. A recent United States Food and Drug Administration evaluation of 12 clinical studies submitted in support of approvals for treatments for MBC suggested that PFS and OS are only weakly correlated (*R*^2^ = 0.079) [9]. This poor correlation may be due, at least in part, to crossover and variations in poststudy anticancer therapies [7].

It can, therefore, be difficult to interpret the true clinical impact of a new agent that improves PFS but not OS, particularly when other treatments with established OS benefits are available [10]. Thus, there is an ongoing debate as to whether benefits in PFS, in the absence of increases in OS, are clinically relevant [11]. By contrast, while less common historically, increases in OS without improvements in PFS have been reported in phase 2 and 3 studies of immunotherapies [12, 13] and with an endothelin receptor antagonist [14, 15] in patients with castration-resistant metastatic prostate cancer, as well as in a phase 3 study comparing antiepidermal growth factor receptor and antivascular endothelial growth factor therapies for metastatic colorectal cancer [16]. Further, it is increasingly questioned whether PFS, as conventionally defined, is a meaningful endpoint in clinical studies of immunotherapies, where a tumor "flare—characterized by active inflammation, enhancement and even potentially increased tumor mass— may be seen before a conventional response has had time to develop [17–19].

Improvements in OS, but not PFS, have been observed in only a few clinical studies of cytotoxic chemotherapy [20–22]. In two recent, large-scale, randomized, phase 3 clinical studies of the nontaxane microtubule dynamics inhibitor eribulin, patients in the eribulin arm appeared to benefit more in terms of OS than PFS [23, 24]. One of these studies (Study 305/Eisai Metastatic Breast Cancer Study Assessing Physician’s Choice Versus E7389 [EMBRACE]) [23] demonstrated a significant increase in the primary endpoint of OS with eribulin compared with treatment of physician’s choice (TPC) in women with MBC who had received between two to five previous chemotherapy regimens, including ≥2 for advanced disease (median 13.1 vs. 10.6 months; hazard ratio [HR] 0.81; 95 % confidence interval [CI] 0.66, 0.99; *p* = 0.041). There was a small but significant difference in favor of eribulin in PFS as assessed by investigator (HR 0.76; 95 % CI 0.64, 0.90; *p* = 0.002), but not independent review (HR 0.87; 95 % CI 0.71, 1.05; *p* = 0.137), likely due to fewer patients being censored with investigator review, resulting in more progression events, compared with independent review. The median PFS values were, however, similar for the independent and investigator reviews [23].

A separate clinical study (Study 301) compared eribulin with capecitabine in patients with MBC who had received up to three prior chemotherapy regimens, including ≤2 for advanced or metastatic disease [24]. This study showed a numerical difference favoring improved OS with eribulin compared with capecitabine; although this difference was not statistically significant (median 15.9 vs. 14.5 months; HR 0.88; 95 % CI 0.77, 1.00; *p* = 0.056). PFS was almost identical in both treatment arms (median 4.1 vs. 4.2 months; HR 1.08; 95 % CI 0.93, 1.25; *p* = 0.30) [24]. Sensitivity analyses adjusting for a crossover effect and postprogression anticancer treatments were consistent with the primary analysis [25], suggesting that neither of these fully explain the apparent discordance between OS and PFS.

In response to the challenges of interpreting PFS or disease-free survival (DFS) data, the European Medicines Agency (EMA) recently published guidelines for consideration when using these endpoints in clinical studies [26]. PFS is a composite endpoint, including the appearance of “new” lesions (i.e., newly detected metastases, in a new or pre-existing metastatic site), progression of existing metastases (i.e., those present at baseline), mixed responses with some lesions increasing in size but others decreasing in size, and death. The EMA guidelines recommend the use of separate analyses that disassemble PFS into specific progression events. Accordingly, we have performed post hoc analyses of data from Study 301 and Study 305/EMBRACE to assess whether the study drugs had different effects on OS according to the type of progression event, and to explore the apparent discordance between OS and PFS.

Our first aim was to investigate whether the type of progression event was predictive for OS in Study 301 and Study 305/EMBRACE. In addition, we investigated whether a modified PFS, “new-metastasis–free survival,” defined as the time from randomization to progression due to a “new” metastasis or death, renders better concordance with OS between the eribulin and control (TPC or capecitabine) arms.

## Methods

The clinical study design and efficacy and safety results of Study 301 [24] and Study 305/EMBRACE [23] have been reported elsewhere. Key eligibility criteria and treatment details are summarized briefly here. All patients gave written, informed consent. Approval was obtained from independent ethics committees and regulatory authorities in participating countries (Appendix I). The studies were conducted in accordance with the World Medical Association (WMA) Declaration of Helsinki (WMA General Assembly, Tokyo, 2004) guidelines of the Committee for Proprietary Medicinal Products/International Conference for Harmonisation/Good Clinical Practice (CPMP/ICH/135/95), and local ethical and legal requirements.

### Clinical study designs

#### Study 301 [24]

Patients who had received up to three prior chemotherapy regimens, including up to two regimens for advanced and/or metastatic disease, were eligible; prior therapy must have included an anthracycline and a taxane. Eligible patients were randomized 1:1 to 1.4 mg/m^2^ eribulin mesylate intravenously (equivalent to 1.23 mg/m^2^ eribulin [as free base]) on days 1 and 8 of a 21-day cycle, or capecitabine 1.25 g/m^2^ orally twice daily on days 1–14 of a 21-day cycle. Patients were stratified by geographic region and human epidermal growth factor receptor 2 status.

#### Study 305/EMBRACE [23]

Patients were required to have received two to five previous chemotherapy regimens, including an anthracycline and a taxane, with at least two regimens for locally recurrent breast cancer or MBC. Eligible patients were randomized 2:1 to eribulin (using the same dose and schedule as in Study 301) or TPC, defined as: any single-agent chemotherapy, hormonal, or biological treatment approved for the treatment of cancer; radiotherapy; or symptomatic treatment alone. Patients were stratified by geographical region, previous capecitabine use, and human epidermal growth factor receptor 2 status.

### Post hoc analysis of PFS and OS

Progression events (determined using Response Evaluation Criteria In Solid Tumors [RECIST] version 1.0) [27] from both clinical studies were categorized as: detection of a new lesion/metastasis (defined as a lesion not identified at baseline); increase in the sum of the longest diameters of pre-existing target lesions (i.e., those present at baseline) or an increase in the size of at least one nontarget lesion reported at baseline; and patients with no reported disease progression that includes death, clinical deterioration, and patients who were censored. If progressive disease was detected objectively (radiologically or by objective clinical measurement of skin lesions), patients in whom a new lesion was detected were classified as such, even if there was also progression of existing lesions. Based on the above classification, new-metastasis–free survival, defined as the time from randomization to death or progression due to a new metastasis (whichever occurred earlier), was used to investigate the treatment effect in the progression type of special interest, and to better understand the potential relationship between disease progression and OS. The time to new metastasis in vital organs (central nervous system [CNS], lungs, or liver) was also calculated because we hypothesized that such metastases may have a particular impact on outcomes.

Independent review of disease progression in the intent-to-treat (ITT) population was used for the primary analyses. Separate analyses were conducted with investigator review (data not shown); however, results by independent review only are presented here because they are considered to be more reliable. Moreover, because PFS from independent reviews showed discordance with OS in both studies, it is further appropriate to explore the reasons of such discordance using the independent review data.

### Statistical analyses

The correlation between progression events and OS was investigated by Cox regression, incorporating the development of a new lesion/metastasis as a time-dependent covariate. OS in the different treatment groups was compared by PFS status using Cox regression and stratified log-rank tests. In the analysis of new-metastasis–free survival, the cumulative incidence competing risk (CICR) method [28] was used to estimate the cumulative probabilities of disease progression due to the development of a new lesion or death, in which progression due to existing lesion was considered as a competing risk. The CICR method (unlike the Kaplan-Meier estimator) does not require any assumption about independence of the competing risks. The independence assumption required by log-rank test implies that at each time point the hazard of developing new metastases or death is the same for patients who were in follow-up and had no event, and for patients who died due to growth of a pre-existing lesion by that time, which cannot be verified in our studies. The unstratified Gray’s test [29] was used to compare the cumulative incidence functions between the two treatment arms in the competing risks setting. Because death or progression due to an existing lesion or nonvital new lesion was censored by definition in the calculation of time to new metastasis in vital organs (i.e., competing risks are not considered), Kaplan-Meier estimator and log-rank tests were used to estimate the treatment difference.

## Results

Study 301 comprised 1102 patients in the ITT population, with 554 in the eribulin arm and 548 in the capecitabine arm. Study 305/EMBRACE comprised 762 patients in the ITT population, with 508 in the eribulin arm and 254 in the TPC arm. Consolidated Standards of Reporting Trials (CONSORT) diagrams for both clinical studies have been published previously [23, 24]. The results described below are based on tumor assessment by independent review; however, similar results were obtained with investigator review (data not shown), unless stated otherwise.

### Study 301

#### Prognostic effects of type of progression

Of the 1102 patients in the ITT population, 1101 had baseline scans, and 420 (38.1 %) patients had disease progression due to the development of new metastases. Patients who were deemed to have tumor progression due to new metastases had an increased risk of death compared with all other patients (n = 682; median OS 13.0 months vs. 17.1 months, respectively), regardless of whether the data were stratified by treatment group or not (HR 1.98 and 1.96; *p* < 0.0001; Tables 1 and 2).

**Table 1:** Disease progression due to new metastasis as a risk factor of overall survival (intent-to-treat population)

	Study 301		Study 305/EMBRACE	
	Stratified by treatment group	Not stratified	Stratified by treatment group	Not stratified
Compared with all other^a^ subjects				
Patients, n	1102		762	
HR (95 % CI)	1.98 (1.71, 2.29)	1.96 (1.70, 2.27)	2.25 (1.79, 2.83)	2.27 (1.80, 2.85)
Wald *p* value	<0.0001	<0.0001	<0.0001	<0.0001
Compared with those whose disease progressed due to an increase in the size of pre-existing lesion(s)				
Patients, n	663		469	
HR (95 % CI)	1.81 (1.54, 2.14)	1.80 (1.53, 2.13)	2.09 (1.63, 2.68)	2.12 (1.65, 2.72)
Wald *p* value	<0.0001	<0.0001	<0.0001	<0.0001
Compared with those with no reported disease progression^b^				
Patients, n	859		500	
HR (95 % CI)	2.20 (1.88, 2.57)	2.16 (1.85, 2.53)	2.61 (2.03, 3.37)	2.60 (2.02, 3.34)
Wald *p* value	<0.0001	<0.0001	<0.0001	<0.0001

Tumor progression due to new metastasis is fitted as a time-dependent variable in the Cox regression model

*CI* confidence interval, *EMBRACE* Eisai Metastatic Breast Cancer Study Assessing Physician’s Choice Versus E7389, *HR* hazard ratio

^a^i.e., progression due to growth of pre-existing lesion(s), or those with no reported disease progression (including death, clinical deterioration or censoring)

^b^Patients with no reported disease progression included death, clinical deterioration, or censoring

**Table 2:** Overall survival for different patient groups in Study 301

	Progression due to new metastases			Progression due to pre-existing lesions			No reported disease progression		
	Eribulin	Capecitabine	Total	Eribulin	Capecitabine	Total	Eribulin	Capecitabine	Total
	(*n* = 216)	(*n* = 204)	(*N* = 420)	(*n* = 131)	(*n* = 112)	(*N* = 243)	(*n* = 207)	(*n* = 232)	(*N* = 439)
Median OS, months (95 % CI)	14.6 (12.4, 17.1)	11.3 (9.2, 13.4)	13.0 (11.6, 14.4)	18.0 (14.7, 19.7)	16.7 (14.2, 19.9)	17.1 (15.5, 19.4)	17.6 (15.4, 20.7)	16.8 (14.3, 19.1)	16.9 (15.7, 18.9)
HR eribulin vs. capecitabine (95 % CI)	0.75 (0.61, 0.93)		–	0.92 (0.69, 1.23)		–	0.91 (0.73, 1.15)		–
*p* value	0.008		–	0.57		–	0.44		–

Progression events were determined by independent review

*CI* confidence interval, *HR* hazard ratio, *OS* overall survival

Similar findings were observed when median OS was compared between patients whose disease progressed due to new metastases and those whose disease progressed specifically due to an increase in the size of pre-existing lesions (*n* = 420 vs. 243, respectively; 13.0 months vs. 17.1 months, respectively), regardless of stratification (HR 1.81 and 1.80; *p* < 0.0001; Tables 1 and 2). Median OS was also shorter for patients who developed new metastases than for those patients with no reported disease progression (which includes death, clinical deterioration and censoring; [*n* = 439], 13.0 months vs. 16.9 months, respectively) irrespective of stratification (HR 2.20 and 2.16, *p* < 0.0001; Tables 1 and 2).

#### Comparison of eribulin versus capecitabine according to progression event

Disease progression due to new metastases occurred in 216 patients (39.0 %) in the eribulin arm compared with 204 patients (37.2 %) in the capecitabine arm. In the eribulin arm, 131 patients (23.7 %) progressed due to an increase in the size of pre-existing lesions, compared with 112 (20.4 %) in the capecitabine arm. Two hundred seven (37.4 %) patients in the eribulin arm, which represented 148 (26.7 %) deaths, and 232 (42.3 %) patients in the capecitabine arm, which represented 179 (32.7 %) deaths, were associated with no reported disease progression.

Median OS for patients whose disease progressed due to new metastases was significantly longer with eribulin than with capecitabine (14.6 vs. 11.3 months; HR 0.75; *p* = 0.008; Table 2). For those whose disease progressed due to an increase in the size of pre-existing lesions, median OS was 18.0 versus 16.7 months (HR 0.92; *p* = 0.57; Table 2). In patients with no reported disease progression, median OS was 17.6 versus 16.8 months (HR 0.91; *p* = 0.44) for eribulin and capecitabine, respectively.

**Fig. 1:**
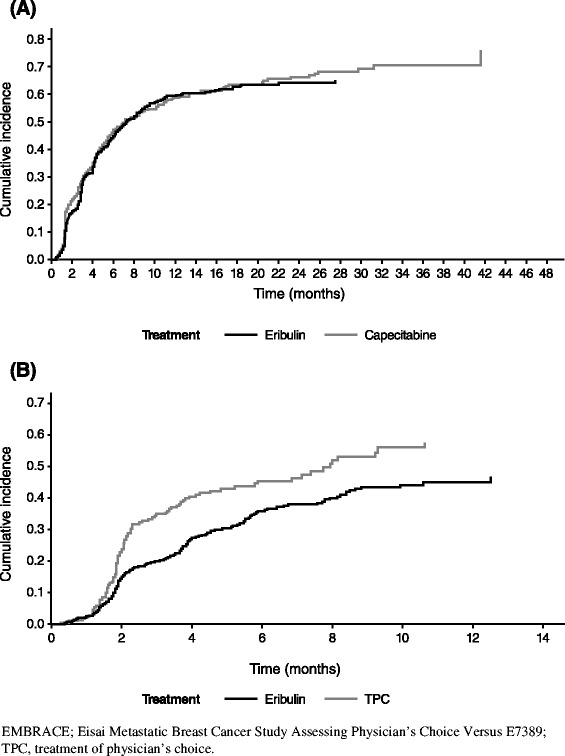
Cumulative incidence function of new metastasis or death in studies (**a**) 301, and (**b**) 305/EMBRACE. The data are based on independent review of the intent-to-treat population.

**Fig. 2:**
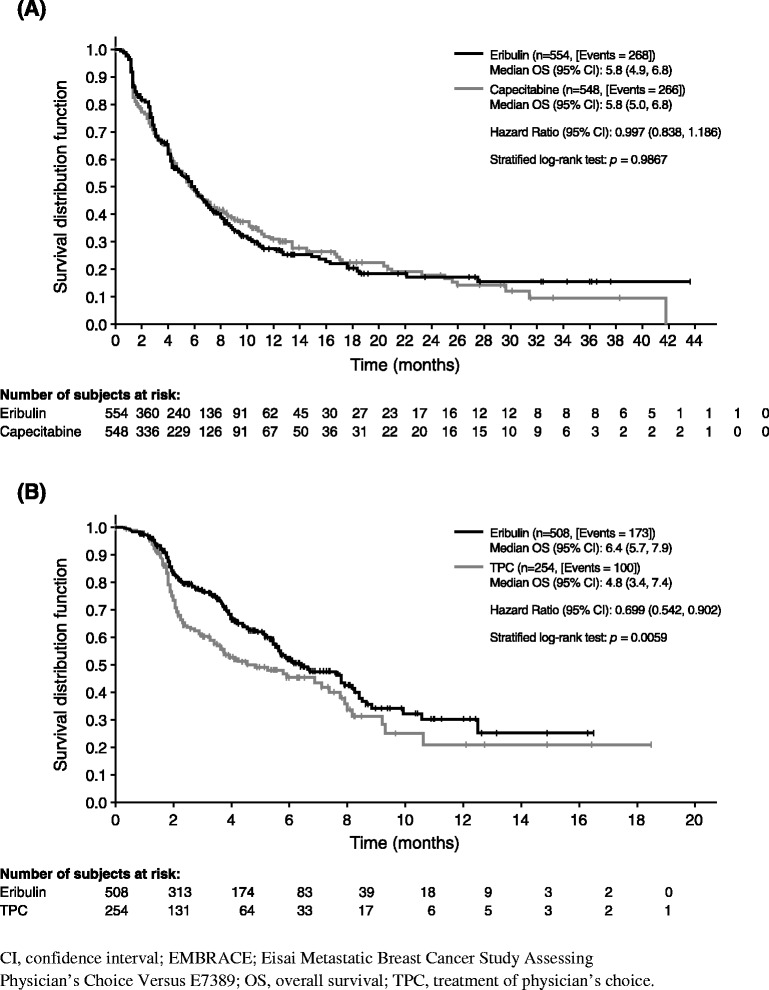
New-metasasis–free survival in studies (**a**) 301 and (**b**) 305/EMBRACE. The data are based on independent review of the intent-to-treat population. New-metastasis–free survival was defined as the time from randomization to death or disease progression due to a new metastasis (whichever occurred earlier).

**Fig. 3:**
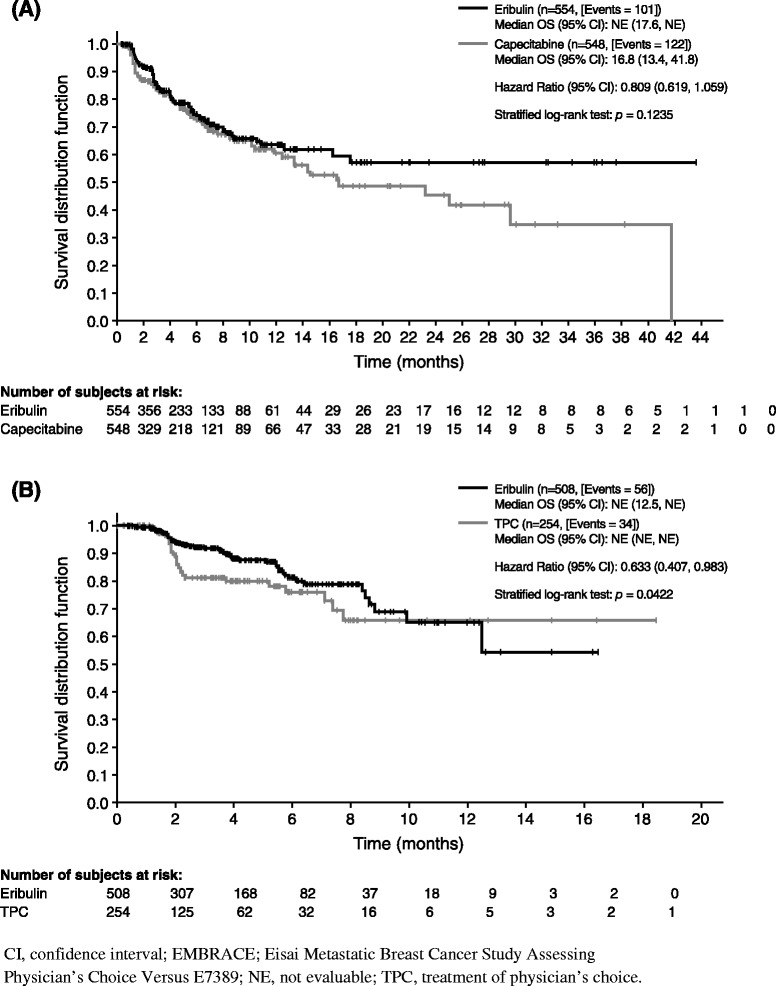
Time to new metastases in vital organs in studies (**a**) 301, and (**b**) 305/EMBRACE. The data are based on independent review of the intent-to-treat population. Vital organs constitute the central nervous system, lungs, or liver.

Cumulative probabilities of new metastasis or death were similar in both arms (Gray’s test *p* = 0.46; Fig. 1a). Sensitivity analysis of new-metastasis–free survival using log-rank test showed similar results (median 5.8 vs. 5.8 months; HR 1.00; *p* = 0.99; Fig. 2a). For patients whose disease progressed due to new metastases, there were no notable differences in the incidence of new metastases in different organs (Table 3). In addition, the time to new metastases in patients whose disease progressed due to new metastases in vital organs (CNS, lungs, or liver) was also not significantly different between the two treatment arms (HR 0.81; *p* = 0.12; Fig. 3a).

**Table 3:** Summary of new metastases by site^a^ based on independent review (intent-to-treat populations)

Parameter	Study 301		Study 305/EMBRACE	
	Eribulin	Capecitabine	Eribulin	TPC
	(*n* = 554)	(*n* = 548)	(*n* = 508)	(*n* = 254)
Patients whose disease progressed owing to new metastases, *n* (%)	216 (39.0 %)	204 (37.2 %)	139 (27.4 %)	68 (26.8 %)
Sites of new metastases^b^, *n* (%)				
Liver	72 (33.3)	80 (39.2)	41 (29.5)	30 (44.1)
Lung	30 (13.9)	33 (16.2)	8 (5.8)	4 (5.9)
Lymph nodes	7 (3.2)	3 (1.5)	16 (11.5)	6 (8.8)
Bone	19 (8.8)	16 (7.8)	7 (5.0)	2 (2.9)
Skin	66 (30.6)	62 (30.4)	3 (2.2)	1 (1.5)
CNS (brain/spine)	4 (1.9)	17 (8.3)	7 (5.0)	0
Breast	12 (5.6)	7 (3.4)	3 (2.2)	0
Chest wall	9 (4.2)	4 (2.0)	25 (18.0)	5 (7.4)
Other	6 (2.8)	13 (6.4)	5 (3.6)	4 (5.9)

*CNS* central nervous system, *EMBRACE* Eisai Metastatic Breast Cancer Study Assessing Physician’s Choice Versus E7389, *TPC* treatment of physician’s choice

^a^Only the first new metastasis observed is recorded. If there were multiple new metastases observed at the same time and all determined as earliest, all new metastasis sites were summarized

^b^Percentages for metastasis sites are based on the number of patients in each study arm whose disease progressed owing to new metastases

#### Comparison of eribulin versus capecitabine according to treatment duration

No significant differences in treatment duration were observed between the patients treated with eribulin versus capecitabine irrespective of the type of progression event subgroup (*p* = ns for all; data not shown).

### Study 305/EMBRACE

#### Prognostic effect of type of progression

The ITT population comprised 762 patients, of whom 760 had baseline scans and 207 (27.2 %) had disease progression due to new metastases. Similar to Study 301, patients with tumor progression due to new metastases were at an increased risk of death compared with all other patients (*n* = 555), whether stratified by treatment group or not (median OS 11.1 vs. 12.5 months, respectively; HR 2.25 and 2.27, respectively; *p* < 0.0001; Tables 1 and 4).

**Table 4:** Overall survival for different patient groups in Study 305/EMBRACE

	Progression due to new metastases			Progression due to pre-existing lesions			No reported disease progression		
	Eribulin	TPC	Total	Eribulin	TPC	Total	Eribulin	TPC	Total
	(*n* = 139)	(*n* = 68)	(*N* = 207)	(*n* = 189)	(*n* = 73)	(*N* = 262)	(*n* = 180)	(*n* = 113)	(*N* = 293)
Median OS, months (95 % CI)	12.3 (9.9, 13.4)	8.4 (7.0, 14.4)	11.1 (8.9, 13.1)	13.0 (11.1, 14.5)	11.1 (8.7, 15.3)	12.2 (11.0, 14.2)	15.8 (12.1, 19.1)	10.7 (9.2, 14.8)	13.0 (11.1, 16.9)
HR eribulin vs. TPC (95 % CI)	0.76 (0.51, 1.12)		–	0.74 (0.50, 1.10)		–	0.77 (0.54, 1.10)		–
*p* value	0.16		–	0.14		–	0.16		–

Progression events were determined by independent review

*CI* confidence interval, *EMBRACE* Eisai Metastatic Breast Cancer Study Assessing Physician’s Choice Versus E7389, *HR* hazard ratio, *OS* overall survival, *TPC* treatment of physician’s choice

The same differences in median OS were also observed for disease progression due to a new metastases compared with progression specifically due to an increase in the size of pre-existing lesions (*n* = 262; median OS 11.1 vs. 12.2 months, respectively), whether stratified by treatment group or not (HR 2.09 and 2.12, respectively; *p* < 0.0001; Tables 1 and 4). Patients in the new metastases subgroup also had an increased risk of death compared with the patients with no reported disease progression (that includes death, clinical deterioration, and censoring), ([*n* = 293]; 11.1 vs. 13.0 months, respectively) irrespective of stratification (HR 2.61 and 2.60; *p* < 0.0001; Tables 1 and 4).

#### Comparison of eribulin versus TPC according to progression event

Disease progression due to new metastases occurred in 139 patients (27.4 %) in the eribulin arm and 68 patients (26.8 %) in the TPC arm; an increase in the size of pre-existing lesions occurred in 189 (37.2 %) and 73 (28.73 %) patients receiving eribulin and TPC, respectively. In the eribulin arm, there were 180 (35.4 %) patients with no reported disease progression, compared with 113 (44.5 %) patients in the TPC arm which comprised 85 (16.7 %) and 61 (24.0 %) deaths in the eribulin and TPC arms, respectively. The proportion of patients whose disease progressed due to new metastases was lower in Study 305/EMBRACE than in Study 301 (27.2 % vs. 38.1 %).

Median OS in the eribulin arm was not statistically different from that in the TPC arm for patients whose disease progressed due to new metastases (12.3 vs. 8.4 months; HR 0.76; *p* = 0.16; Table 4), for those whose disease progressed due to an increase in the size of pre-existing lesions (13.0 vs. 11.1 months; HR 0.74; *p* = 0.14; Table 4) or those with no reported disease progression (15.8 vs. 10.7 months; HR 0.77; *p* = 0.16; Table 4). Although, in the investigator review analysis, the difference in median OS with eribulin compared with the TPC arm in patients with no reported disease progression reached statistical significance (16.1 vs. 9.9 months; HR 0.66; *p* = 0.03).

New-metastasis–free survival was longer in the eribulin arm compared with the TPC arm, as indicated by the lower cumulative incidence rate curve of the eribulin arm (Fig. 1b). The differences in cumulative functions between the two arms were statistically significant (Gray’s test, *p* = 0.0017). Sensitivity analysis of new-metastasis–free survival using log-rank test showed similar results (median 6.4 vs. 4.8 months; HR 0.70; *p* = 0.006; Fig. 2b).

There were again some differences in the sites of new metastases between the eribulin and TPC arms (Table 3), but these were modest and not consistent with Study 301, with the exception of patients in the eribulin arm appearing more likely to develop lymph node, breast, and chest wall metastases, and patients in the comparator arm appearing more likely to develop liver and “other” metastases in both Study 305/EMBRACE and Study 301. The time to new metastases in vital organs favored eribulin over TPC (HR 0.63; *p* = 0.04; Fig. 3b).

#### Comparison of eribulin versus TPC according to treatment duration

The patients whose disease progressed due to the development of a new metastasis were treated with eribulin for a significantly longer duration than those receiving capecitabine (median 139 vs. 68 days; *p* < 0.001). Similar results were also observed in the subgroups of eribulin and capecitabine patients whose disease progressed due to an existing lesion (median: 189 vs. 73 days, respectively; *p* < 0.01) and in those with no reported disease progression (median 174 vs. 107 days, respectively; *p* < 0.01).

## Discussion

This analysis is, to our knowledge, the first to focus specifically on new metastases as the cause of disease progression and to demonstrate that patients whose disease progresses due to the development of new metastases have worse OS time than those whose disease progresses due to growth of pre-existing metastases and in comparison with patients with no reported disease progression (the latter includes patients who died for reasons other than the development of new metastasis or growth of pre-existing lesions, patients who experienced clinical deterioration and those that were censored). This confirms the clinical impression that patients who develop new metastasis may have a worse prognosis. Current RECIST criteria do not, however, distinguish between the different progression events.

Only a limited number of published clinical studies have described how different progression events affect OS, and their findings have varied. A post hoc analysis of progression events in 246 patients with metastatic renal cell cancer treated with everolimus in the REnal Cell cancer treatment with Oral RAD001 given Daily (RECORD-1) clinical study showed that the development of new lesions and growth of nontarget lesions were both predictive of worse OS (*p* < 0.001) in univariate analyses [30]. In a multivariate analysis, nontarget progression retained statistical significance (*p* = 0.005), but new metastatic lesions had only borderline significance (*p* = 0.053) [30]. In a metastatic colorectal cancer clinical study, the appearance of new lesions or unequivocal progression of nonmeasurable lesions both carried a worse prognosis (regarding survival) than other measures of progression; this analysis was, however, restricted to assessment of the first on-study response assessment [31]. The impact of different time-dependent measures of response on OS was recently described in a series of breast, lung, and colorectal cancer studies [32]. In these analyses, worse OS was significantly associated both with the development of new metastatic lesions (*p* < 0.001) and progression of nontarget lesions (*p <* 0.001).

In contrast, a preliminary report from the phase 3 Intergroup study N9741 in 726 patients with advanced colorectal cancer whose disease progressed on chemotherapy found that individuals whose disease progressed due to the development of new metastases had similar median OS outcome as patients whose disease progressed in pre-existing lesions [33].

Our analyses demonstrate that progression due to new metastases is, indeed, associated with significantly worse OS in patients with pretreated MBC. This finding has important potential implications. In the context of clinical studies, it is possible that one type of progression event may be more predictive of OS than other types of progression events. The difference in prognosis, according to the type of progression, might also reflect eligibility criteria: if, for example, the desire is to select patients with a better prognosis, those progressing with new metastases might be excluded. In clinical practice, these data may also help to inform oncologists and their patients when discussing future treatment options and prognosis.

Our second objective was to investigate whether the different types of progression explain the greater impact of eribulin on OS than on PFS. “New-metastasis–free survival,” a modified PFS that treats death and new metastases (i.e., the more aggressive type of progression) as the event of interest and progression due to existing lesion as a competing risk, was compared between eribulin and the comparator arms. Using the CICR method, new-metastasis–free survival was longer in the eribulin arm than in the comparator arm for Study 305 but not in Study 301. The log-rank test for comparing hazards of new metastases or death also resulted in a *p* value of 0.006 in the sensitivity analysis for Study 305. Taken together, our results suggest that the development of new metastases is a risk factor for worsening OS and that the lower incidence rates of new metastasis/death in the eribulin arm may explain, at least in part, the OS benefit associated with eribulin in Study 305. Additionally, in Study 305, patients in the eribulin arm were treated for longer than those in the TPC arm irrespective of the type of progression event, which may also have contributed to better OS.

Although in Study 301 there was no difference in the new-metastasis–free survival between the treatment arms, the different types of progression events may provide some explanation for the OS differences observed between treatment arms. The modest increase in OS with eribulin compared with capecitabine in Study 301 was primarily driven by a significant improvement in OS in the new metastasis subgroup, whereas in Study 305 a similar but nonsignificant increase in OS was observed in the eribulin arm in all three “progression” subgroups. In both clinical studies, the proportion of patients with no reported disease progression was lower in the eribulin arm than in the comparator arm (37.4 % vs. 42.3 % in Study 301, and 35.4 % vs. 44.5 % in Study 305).

Thus, new-metastasis–free survival alone cannot account for the observed discordance between PFS and OS; however, a possible explanation for it may be attributed to the recently identified effects of eribulin on the tumor microenvironment. Recent data in human breast cancer models indicate that eribulin may remodel the tumor microenvironment and vasculature, which may improve its antitumor activity and drug delivery compared with other cytotoxic agents [34]. These changes may also potentially impact on the effects of concomitant and/or subsequent anticancer therapies; further studies may be able to substantiate this hypothesis.

One limitation of our analysis of the impact of the development of new metastases on OS is that it was post hoc rather than preplanned. Further, this analysis was not powered for the subgroups reviewed; subsequently, no definitive conclusions can be drawn. These analyses were, however, prompted by the EMA guidelines. It may also be questioned whether our findings can be generalized to the broader population of patients with MBC. Study 301 and Study 305/EMBRACE were, however, both large well-designed phase 3 studies, with approximately a third (33.6 %) of the patients developing new metastases and more than a quarter (27.1 %) experiencing progression due to growth of pre-existing lesions. Further, although over half of patients (57.0 %) were treated with a single cytotoxic agent (i.e., eribulin), the remainder received a range of cytotoxic agents used to treat MBC. Finally, our finding of the association between the development of new metastases and worse OS was consistent across both clinical studies. These retrospective findings warrant further investigation in phase 3 MBC datasets as well as prospective evaluation.

## Conclusions

Patients with MBC who develop tumor progression with new metastases have a worse prognosis than patients whose disease progresses due to growth of pre-existing lesions and compared with patients with no reported disease progression. This finding has potentially important implications both for the interpretation of clinical study results, especially when PFS is the primary endpoint, and for clinical practice. “New-metastasis–free survival” may be a useful complement to PFS in understanding the dynamic between disease progression and survival. The relationship between the different progression events and OS warrants further investigation.

## Abbreviations

CI: confidence interval
CICR: cumulative incidence competing risk
CNS: central nervous system
CONSORT: Consolidated Standards of Reporting Trials
CPMP: Committee for Proprietary Medicinal Products
DATECAN: Definition for the Assessment of Time-to-event Endpoints in CANcer trials
DFS: disease-free survival
EMA: European Medicines Agency
EMBRACE: Eisai Metastatic Breast Cancer Study Assessing Physician’s Choice Versus E7389
HR: hazard ratio
ICH: International Conference for Harmonisation
ITT: intent-to-treat
MBC: metastatic breast cancer
ORR: objective response rate
OS: overall survival
PFS: progression-free survival
RECIST: Response Evaluation Criteria In Solid Tumors
RECORD-1: REnal Cell cancer treatment with Oral RAD001 given Daily
TPC: treatment of physician’s choice
WMA: World Medical Association
